# Ambient-Dried Silica Xerogels with Enhanced Strength and Thermal Insulation via Calcium Ion-Glycerol Synergistic Crosslinking

**DOI:** 10.3390/gels11060462

**Published:** 2025-06-16

**Authors:** Xiaoyu Xie, Zilin Zhu, Yu Meng, Lijia Wang, Fuquan Zhao, Lingqing Chen, Lijie Jiang, Ming Yan, Xiaofan Zhou

**Affiliations:** 1 Jiangsu Co-Innovation Center of Efficient Processing and Utilization of Forest Resources, National Engineering Research Center of Biomaterials, Nanjing Forestry University, Nanjing 210037, China; 2 Jiangsu Provincial Key Lab of Pulp and Paper Science and Technology, College of Light Industry and Food, Nanjing Forestry University, Nanjing 210037, China; 3 Jiangsu Engineering Research Center of Bamboo and Wood Carbon Fixation Materials and Structures, Nanjing Forestry University, Nanjing 210037, China; 4 Zhejiang Jinchang Special Paper Co., Ltd., Quzhou 324000, China; 5 Sherwin-Williams South China Technology Center Co., Ltd., Foshan 528306, China; 6 Guangdong Paper Industry Research Institute Co., Ltd., Guangzhou 510300, China

**Keywords:** silicon-calcium xerogel, sol-gel processes, high strength, atmospheric drying

## Abstract

Despite their high porosity and wide applicability, silica xerogels face mechanical strength limitations for high-performance applications. This study presents an ambient-pressure sol-gel strategy utilizing calcium-glycerol synergy to produce robust xerogels with enhanced properties. Physicochemical analyses reveal that controlled Ca^2+^ incorporation (optimal at 6 wt.%) accelerates gelation kinetics while establishing a hybrid network through ionic complexation and hydrogen bonding. The resulting xerogels achieve exceptional compressive strength (30.8 MPa) while maintaining uniform mesoporosity (50–90 nm pore size). Remarkably, the as-prepared silica xerogels demonstrate outstanding thermal insulation, maintaining a 220 °C temperature differential in 300 °C environments. These results prove that the ambient-pressure sol-gel strategy utilizing calcium-glycerol synergy can enhance the mechanical performance and thermal insulation performance of silica xerogels with the dual actions of Ca^2+^-induced network reinforcement via silanol coordination and glycerol-mediated stress relief during ambient drying. Overall, this work can offer a scalable, energy-efficient approach to produce high-performance silica xerogels with huge potential in building envelopes and aerospace systems.

## 1. Introduction

Silica xerogel is a kind of porous material prepared by the sol-gel method [1,2], which possesses a high specific surface area, controllable pore structure, low density, and excellent thermal and chemical stability [3,4,5,6]. These advantageous properties enable their widespread application in catalysis [7], adsorption [8,9], separation [10], sensors [11], drug delivery [12], thermal insulation [13,14,15], and environmental protection [16]. However, the large-scale industrial production of silica xerogels remains relatively restricted due to the high manufacturing cost of silica aerogels and the laborious methods for drying. It is worth noting that the drying process is particularly critical during the preparation of silica xerogels [17,18]. The supercritical drying (SCD) method is the most common method for preparing silica xerogels. In contrast, the ambient pressure drying (APD) method possesses a series of advantages, such as low cost, high safety, and fast production [19,20]. Atmospheric pressure drying is a simple process for aerogel synthesis with low equipment requirements and relatively low drying costs. Therefore, optimized sol-gel and APD processes are expected to transform silica aerogel products from good but expensive into excellent and inexpensive, which may significantly promote their practical applications [6,21].

However, silica xerogels fabricated via conventional atmospheric pressure drying still exhibit several critical limitations, most notably their inherently poor mechanical strength [22,23]. Conventional silica xerogels typically exhibit pronounced brittleness, rendering them susceptible to fracture or structural collapse under mechanical or thermal stress [24]. This inherent fragility severely restricts their applicability in high-performance domains demanding exceptional durability and long-term stability. Therefore, the development of a robust synthesis strategy for silica xerogels with enhanced mechanical strength holds substantial scientific significance and offers promising potential for practical applications in demanding environments. Recently, many researchers have carried out a series of works in this area. Wassgren et al. successfully fabricated silica-embedded (SE) xerogels, demonstrating a maximum compressive strength of 940 kPa [25]. Huang et al. reported lignocellulosic xerogels with a mechanical strength of only 782 kPa [26], which falls significantly below the minimum requirements for most industrial applications. This mechanical deficiency likely stems from insufficient cross-linking density and weak skeletal architecture, which fail to effectively dissipate applied stresses, ultimately leading to structural collapse under load. To address these limitations, substantial research has been devoted to improving the mechanical robustness of xerogels while preserving their advantageous thermal and structural properties. Recent advances in silica xerogel reinforcement strategies primarily focus on the following two approaches. On one hand, organic-inorganic hybridization via co-condensation of organosilanes with inorganic precursors to form reinforced hybrid networks [27,28,29,30]. On the other hand, crosslinking enhancement through ionic modifiers (e.g., Ca^2^⁺, Al^3^⁺) is introduced via ion exchange or covalent bonding [2,31,32,33]. However, both approaches face certain limitations. While the former demonstrates improved toughness through molecular-scale hybridization, it often compromises thermal stability above 300 °C due to organic component degradation. The latter approach, though effective in strengthening the silica network, frequently results in heterogeneous pore structures and drying-induced collapse due to uncontrolled crosslinker distribution. Consequently, developing a scalable synthesis strategy that simultaneously optimizes mechanical robustness, thermal insulation performance, and structural uniformity remains a critical challenge in the preparation of silica xerogels.

Herein, this study proposes an innovative dual-modification strategy for fabricating high-performance silica xerogels through the synergistic combination of controlled calcium ion (Ca^2^⁺) concentration and glycerol incorporation in the sol-gel system. Departing from conventional ionic crosslinking approaches that typically yield heterogeneous networks or structural damage, the synergistic use of Ca^2^⁺ ions and glycerol enables the formation of a uniform three-dimensional crosslinked framework. First, Ca^2^⁺ ions act as dynamic crosslinkers, forming coordination complexes with surface silanol groups to enhance network connectivity. Meanwhile, glycerol serves as both a polyol-based drying control chemical additive and network modifier, effectively mitigating capillary stresses during solvent evaporation, thereby minimizing pore collapse and cracking. Furthermore, the cooperative hydrogen bonding between glycerol hydroxyl groups and silicate species generates an organic-inorganic hybrid network with exceptional mechanical resilience. The optimized xerogels demonstrate remarkable property enhancements in this work, achieving compressive strengths up to 30.8 MPa while maintaining ultralow thermal conductivity. Overall, this facile, cost-effective approach not only overcomes the traditional scalability limitations of silica xerogels but also establishes a versatile strategy for developing next-generation thermal insulation materials suitable for building envelopes, aerospace thermal protection systems, and sustainable packaging solutions.

## 2. Results and Discussion

### 2.1. Gel Process

**Figure 1:**
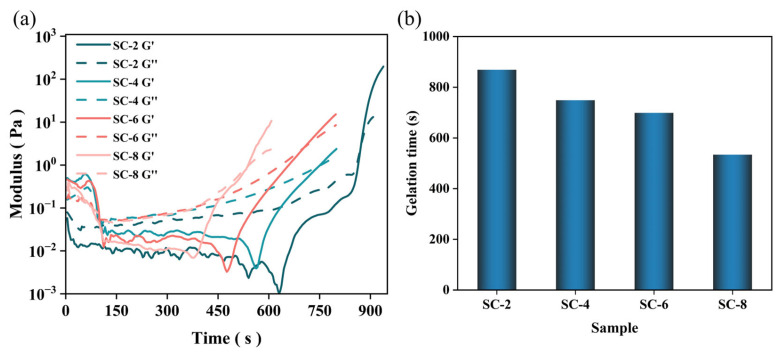
(**a**) Storage modulus (G′) and loss modulus (G″) of samples at 25 °C, 0.10% strain magnitude, and 1.0 Hz. Samples were prepared with 2–8% Calcium ion content. (**b**) The gelation time indicates the time when G′ = G″.

Figure 1 demonstrates the pronounced impact of calcium ion concentration on gelation kinetics and rheological evolution. A systematic reduction in gel time was observed, decreasing from 870 s to 535 s as Ca^2+^ content increased from 2% to 8%, which indicates that calcium ions can significantly promote the formation of the gel network. This acceleration effect was further corroborated by rheological measurements, where both storage (G′) and loss (G″) moduli exhibited concentration-dependent gelation behavior. Notably, the crossover point (G′ = G″) occurred substantially earlier at higher Ca^2+^ concentrations, with 8% CaCl_2_ samples reaching gelation 40% faster than their 2% counterparts [34]. The observed rheological transition is typically characterized by three stages: (1) initial liquid-like behavior (G″ > G′), (2) viscoelastic transition at gel point (G′ = G″), and (3) final solid-like response (G′ > G″) [35]. This progression signifies the development of a percolating network structure, where Ca^2+^ ions act as efficient crosslinkers through bridging between silica nanoparticles. The concentration-dependent acceleration suggests that higher Ca^2+^ availability increases crosslinking density, thus leading to enhanced interparticle connectivity, more rapid stress-bearing network formation, and improved structural integrity at early stages. This is a desirable characteristic for applications where rapid gelation and early mechanical strength are required, such as in thermal insulation materials or other industrial processes that demand quick solidification. Moreover, the mechanical implications of this accelerated gelation will be further explored in subsequent compression testing and structural characterization.

**Figure 2:**
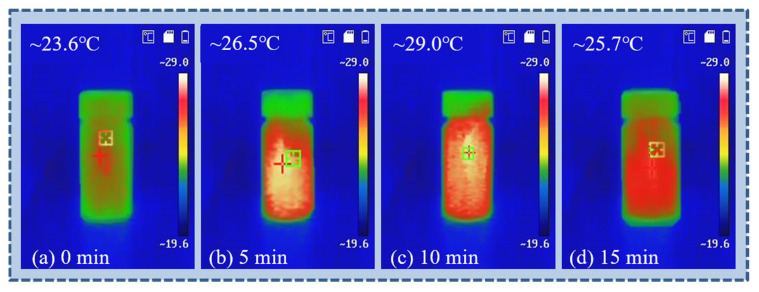
Infrared imaging of silica sol under initial conditions (**a**), 5 min (**b**), 10 min (**c**), and 15 min (**d**).

Upon the addition of calcium ions into the silica sol, a rapid temperature increase was observed, rising from 23.6 to 29.0 °C within 10 min, as captured by infrared thermal imaging (Figure 2). This exothermic behavior is attributed to the hydration of Ca^2^⁺ ions and their coordination with silanol and silicate groups in the sol system. The release of hydration and complexation heat indicates active ionic interactions, thereby facilitating the early formation of the silica network and enhancing the uniformity of gelation. This thermal response supports the role of calcium ions not only as structural crosslinkers but also as reaction activators in the sol-gel process.

### 2.2. Structural and Morphological Characterization of SC Xerogel

**Figure 3:**
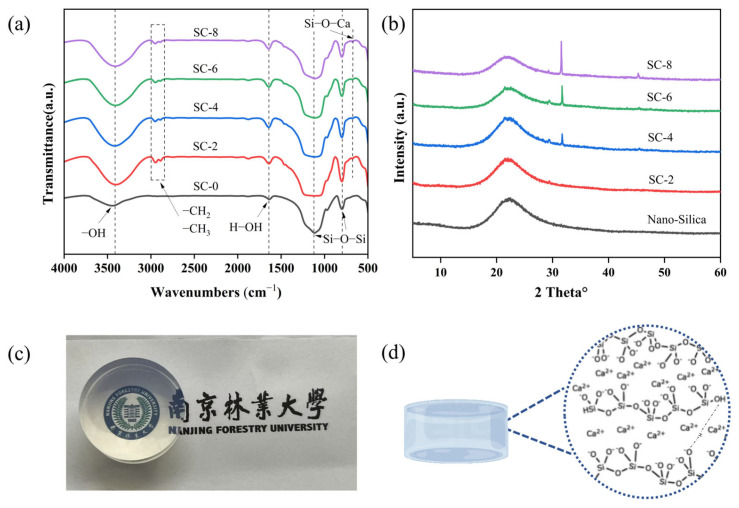
(**a**) FTIR Spectrum results of nano-silica and SC-xerogel. (**b**) XRD pattern of nano-silica and SC-xerogel (**c**) Morphology diagram of SC-6 xerogel (**d**) Schematic diagram of gel network.

As shown in Figure 3a, the spectrum of nano-silica and SC 2–SC 8 xerogel exhibits a wide and strong absorption peak at 3000–3600 cm^−1^, attributed to the stretching vibration of O–H [36]. The characteristic peaks at 1640 cm^−1^ were attributed to bending vibration and rotational motion of H–O–H in the crystal water [37,38]. Two small peaks appear at 2889 cm^−1^ and 2951 cm^−1^, which correspond to the symmetric pattern of C–H stretching vibration and asymmetric pattern of C-H stretching vibration [39]. For silica aerogel, the peaks at 798 cm^−1^ and 1110 cm^−1^ corresponded to the symmetric and antisymmetric stretching vibration of Si–O–Si, respectively [40]. The peak at 955 cm^−1^ belonged to the bending vibration absorption peak of Si–OH, indicating that a successful chemical network was developed. In the infrared spectrum, a small and subtle peak is observed at 675 cm^−1^, along with the broadening of the peak around 1100 cm^−1^, suggesting significant structural or chemical interactions. The peak at 675 cm^−1^ typically corresponds to vibrations associated with Si–O–Ca bonding, indicating potential interactions between calcium (Ca) and silicon dioxide (SiO_2_). This peak implies that a Si–O–Ca linkage might have formed, which can result from a reaction or interaction between calcium compounds (e.g., calcium chloride) and silicon dioxide. The broadening of the peak at around 1100 cm^−1^, which generally corresponds to the Si-O-Si stretching vibration in silicon dioxide, further supports the idea of an interaction [41]. The broadening could be caused by perturbations in the Si-O-Si network, which may result from the formation of a Si–O–Ca bond. This alteration in the Si-O-Si stretching mode typically occurs when the local structure becomes disordered, as is the case when calcium ions disrupt the symmetry of the silica network, leading to a broadened infrared absorption feature [42].

Figure 3b shows the preparation of silica gels with different calcium ion concentrations by the sol-gel method. XRD analysis results show that the effect of different calcium ion concentrations on the gel has a single wide peak at about 23°, which is a typical feature of amorphous silica [43]. A set of obvious diffraction peaks at 31.6° was observed, corresponding to the (111) reflections of CaCl_2_ [44]. This result confirms the effective dispersion of calcium chloride in the gel network and promotes the cross-linking and condensation of silica nanoparticles through ion exchange. In situ rheological analysis (Figure 3) reveals that Ca^2^⁺ accelerates gelation by shortening the induction time from 15 min (2% Ca^2^⁺) to 4 min (8% Ca^2^⁺). The power-law relationship between G′ and Ca^2^⁺ concentration (G′ ∝ [Ca^2^⁺]) indicates a non-linear reinforcement effect, suggesting that Ca^2^⁺ not only bridges Si–OH but also modifies the silica condensation kinetics, as reported by [45].

Si–OH + Si–OH → Si–O–Si + H_2_O (1)

2Si–OH + Ca^2+^ → Si–O–Ca–O–Si + 2H^+^ (2)

The silica nanoparticles provide the main skeleton structure of the gel. The high specific surface area and the active surface of nanoparticles make it easy to form three-dimensional networks. Calcium chloride as an electrolyte can promote the aggregation and crosslinking of silica nanoparticles. Under the action of calcium chloride, the silica nanoparticles provide the main skeleton structure of the gel. The high specific surface area and the active surface of nanoparticles make it easy to form three-dimensional networks. Calcium chloride as an electrolyte can promote the aggregation and crosslinking of silica nanoparticles. Ion exchange between Ca^2^⁺ and silanol groups (Si–OH) forms Si–O–Ca–O–Si bridges, as evidenced by the FTIR peak at 675 cm^−1^ (Figure 3). This bonding increases the network connectivity, with the optimal Ca^2^⁺ content (6 wt.%) achieving a balance between crosslinking density and structural homogeneity. Excessive Ca^2^⁺ (8 wt.%) induces localized aggregation, explaining the 12% strength reduction in SC-8.

### 2.3. Microstructure Characterization

**Figure 4:**
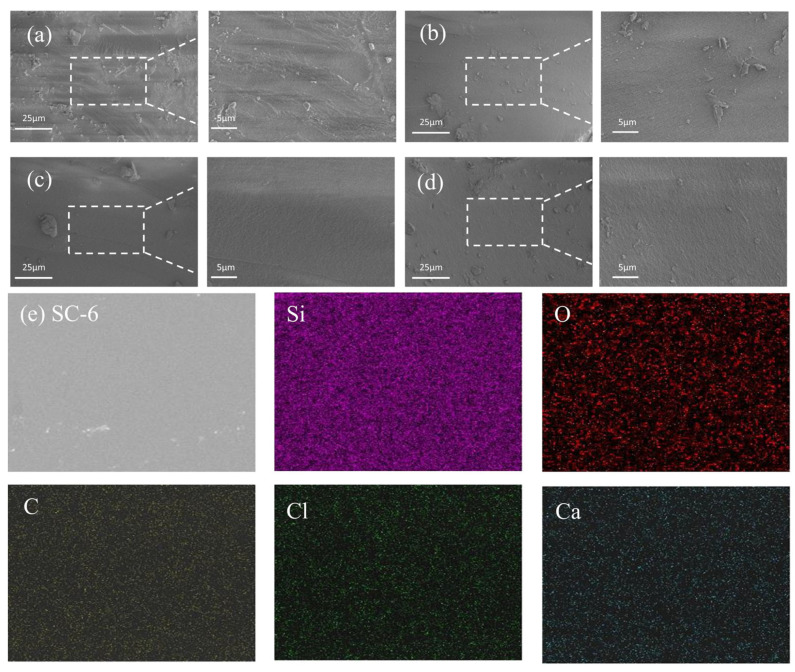
SEM images of SC xerogel. (**a**) SC-2, (**b**) SC-4, (**c**) SC-6, (**d**) SC-8, (**e**) Elemental mapping of silicon, oxygen, carbon, chlorine, and calcium.

To further investigate the structure of xerogels, the microstructures of xerogels were determined by scanning electron microscopy (SEM) as shown in Figure 4a–d. The surface of the sample is smooth, without obvious cracks or agglomeration phenomena, indicating that the gel formation and drying process during the preparation were well controlled. When the Ca^2+^ content was 6 wt.%, the surface of the xerogel was remarkably smooth, indicating a more complete three-dimensional structure of the xerogel. It can be predicted that the optimal addition amount of calcium ions can be determined as 6 wt.% based on the SEM results, and it should be further optimized according to the mechanical property tests. To investigate the elemental distribution of the SC xerogels, a specific area of the surface was chosen for energy spectrum (EDS) analysis. The elemental mapping by the EDS analysis (Figure 4e) was used to detect the presence of Si, O, C, Ca, and Cl elements, and their good distribution in the xerogel Sample SC-6 could be seen. This result further confirms the uniform dispersion of calcium chloride and glycerol in the network, which provides a structural basis for the improvement of its mechanical properties.

### 2.4. Nitrogen Sorption Analysis

**Figure 5:**
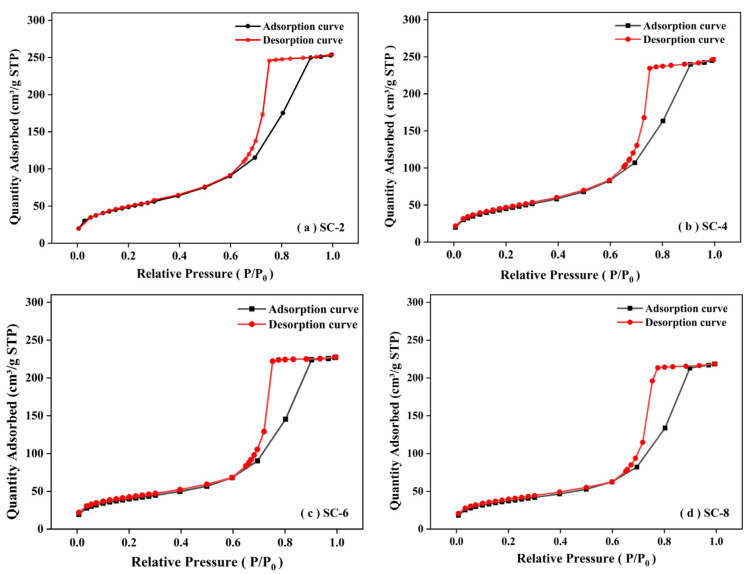
Nitrogen adsorption (black line) and desorption (red line), and pore diameter distribution of (**a**–**d**).

N_2_ adsorption–desorption isotherms of various xerogels, along with their pore size distribution, are portrayed in Figure 5a–d. Surface area and pore volume are listed in Table 1. It could be seen that both SC xerogels had type V isotherms and type H2 hysteresis loops [45,46]. Type H2 is associated with ink-bottle-shaped pores with poor connectivity. The main pore space was occupied by mesopores and macropores [47]. The specific surface area and pore volume data indicate that, with an increase in calcium content, the specific surface area of the xerogel gradually decreases, yet it still maintains a high level (Table 1). This result is consistent with the structural changes observed by SEM, indicating that the introduction of calcium chloride has a certain effect on the pore structure of the material while improving the mechanical strength. Nevertheless, the specific surface area of all samples remained at a high level (>130 m^2^/g). The BET analysis reveals that all xerogels possess high pore volumes ranging from 74.66 to 75.15 cm^3^/g, indicating well-preserved porous networks after ambient drying. While the specific surface area gradually decreases from 177.26 to 130.80 m^2^/g with increasing calcium ion concentration, this trend can be attributed to enhanced crosslinking and structural densification, which lead to reduced microporosity and improved mechanical strength. Despite the reduction in surface area, the large and stable pore volumes ensure effective thermal insulation by suppressing heat transfer pathways. These results demonstrate that the controlled addition of calcium ions, combined with glycerol-assisted drying, allows the formation of mechanically robust xerogels without compromising their thermal performance.

### 2.5. Mechanical Properties of Silicon-Calcium Xerogels

**Figure 6:**
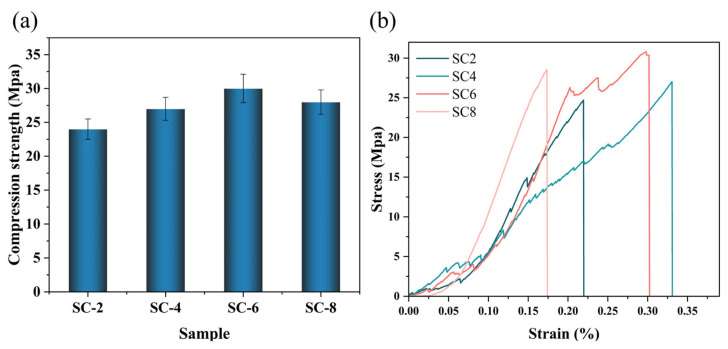
(**a**) Compression strength of silicon-calcium xerogel. (**b**) Stress-strain curve.

As shown in Figure 6, the compressive strength first increased and then slightly decreased with the increase of Ca^2^⁺ content, reaching the peak at 6 wt.% (approximately 30.8 MPa for the SC-6 sample). It is indicated that at an appropriate concentration, Ca^2^⁺ effectively promotes the cross-linking between nano-silica particles, constructing a dense and robust three-dimensional network structure. Calcium ions, as multivalent cations, can coordinate with the hydroxyl groups on the surface of silicon dioxide, enhancing the physical-chemical linkages between particles. Under normal pressure-drying conditions, a strong network structure effectively suppresses dry shrinkage and crack formation, thereby improving mechanical properties. However, an excessively high concentration of Ca^2^⁺ (such as 8 wt.%) may lead to local agglomeration or excessive cross-linking of the gel network, which instead reduces the structural uniformity and thereby slightly lowers the strength. Compared with other articles [48,49], the silica xerogel prepared in this article is significantly superior to the conventional SiO_2_ xerogel and most composite aerogels in the literature.

The remarkable mechanical performance of the SC-6 xerogel stems from the synergistic crosslinking mechanism established by Ca^2^⁺ ions and the polyol network modifier, glycerol. Calcium ions facilitate ionic coordination bridges (Si–O–Ca–O–Si), significantly increasing network rigidity and density. Concurrently, glycerol not only reduces drying-induced capillary stress but also engages in hydrogen bonding with silica surfaces, acting as a flexible binder to dissipate localized mechanical stress. This dual mechanism results in improved crack resistance and load-bearing capacity during ambient drying, thereby achieving compressive strengths exceeding 30 MPa—over an order of magnitude higher than conventional xerogels.

The xerogel density prepared in this manuscript is 1.44 g/cm^3^. It is much higher than the density of conventional aerogel, which is between 0.25 and 0.35 g/cm^3^. Traditional gels are usually dried completely by supercritical drying or freeze-drying methods. They have a relatively low density and strength because their porous structure cannot resist the collapse of voids caused by external stress. Secondly, the relatively weak intermolecular forces between the organic phase and the inorganic phase may cause the inorganic colloidal particles to function like defects in a hybrid structure, thereby reducing the compressive strength. The silica-calcium dry gel prepared in this paper, compared with other xerogels and aerogels, has a high inorganic content. Meanwhile, it adopts the atmospheric pressure drying technology, contains trace amounts of moisture, and has hydrogen bonding effects. Meanwhile, the gel skeleton structure is continuous and strong, sufficient to resist external forces in conventional application scenarios.

### 2.6. The Thermal Properties of Silicon-Calcium Xerogel

**Figure 7:**
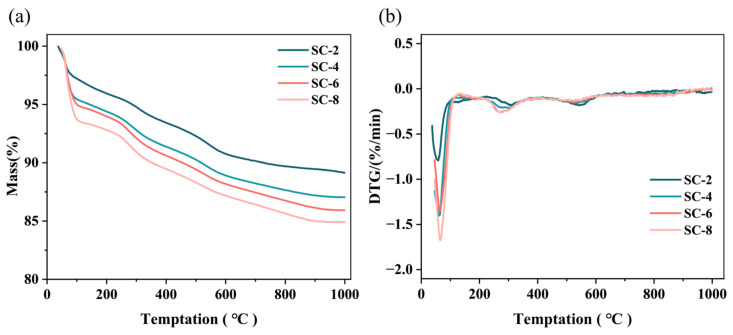
TG (**a**) and DTG (**b**) curves of silicon-calcium xerogel.

The thermal stability of the xerogel was evaluated using thermogravimetric analysis (TGA), as shown in Figure 7. All samples showed significant mass loss in the range of 50–100 °C, which was attributed to the evaporation of adsorbed and crystalline water in the material [2]. Although the sample has undergone drying, it still retains some free water and bound water. The mass loss of the sample at 250–400 °C may be due to the decomposition of organic matter, and the mass is about 5 wt.%, which is consistent with the quality of the raw material. With the increase in temperature, the mass loss of xerogel gradually slowed down, indicating that the material can still maintain good structural stability at high temperatures. The residual mass of the sample at 1000 °C is between 85 wt.% and 90 wt.%, indicating that the sample has a certain temperature resistance and has great potential in thermal insulation [18]. This high residual mass confirms that the three-dimensional SiO_2_–Ca^2^⁺ network remains structurally intact at elevated temperatures, with minimal degradation. The results suggest that the ionic crosslinking between calcium ions and silanol groups provides effective thermal reinforcement, preventing collapse of the silica matrix under thermal stress.

**Figure 8:**
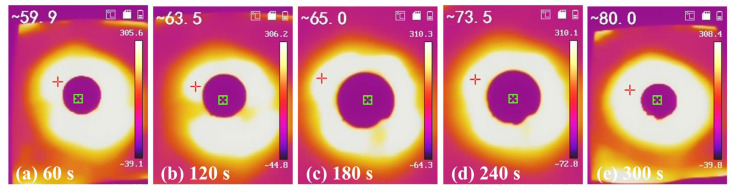
Infrared imaging of SC-6 xerogel under 300 °C.

As shown in Figure 8, the silicon-calcium xerogel response to heat exposure was evaluated by placing it in a 300 °C furnace, simulating high-temperature conditions. After just 1 min, the surface temperature of the xerogel reached only 59.9 °C, significantly lower than the furnace temperature. Over the subsequent 4 min, the surface temperature increased gradually, but after 5 min, the gel’s temperature only reached 80 °C. This temperature rise is far lower than the 300 °C environment, demonstrating the gel’s exceptional thermal insulating capabilities [13]. The excellent thermal resistance is primarily attributed to the material’s low thermal conductivity, which arises from its highly porous structure. The interconnected pores effectively trap air and suppress heat transfer through conduction and convection. Additionally, the incorporation of calcium ions enhances the mechanical stability of the silica network, allowing the material to maintain its structural integrity under elevated temperatures. These results underscore the gel’s potential to be used in various thermal insulation applications, including energy-efficient building materials, industrial heat management, and fire-resistant materials. Its lightweight nature, combined with its high thermal resistance, makes it a promising candidate for the next generation of thermal insulating materials.

**Figure 9:**
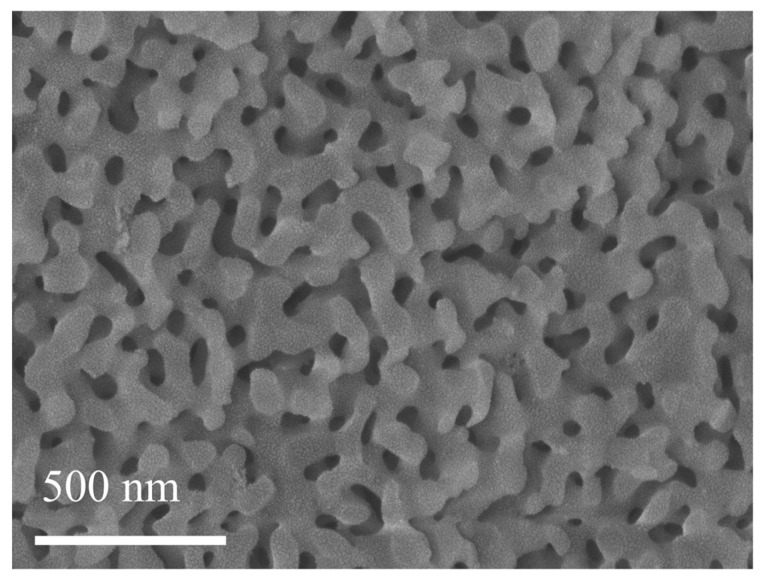
SEM image of the SC-6 sample after complete drying.

The scanning electron microscope (SEM) image of the SC-6 sample (Figure 9), subjected to heating at 300 °C, reveals a porous structure composed of nanoparticles. The microstructure shows that the gel is made up of well-dispersed nano-sized particles, forming a highly porous network [3]. The image indicates that the pore distribution is uniform throughout the gel, with pores being evenly spread across the entire surface area. The observed uniformity in the pore structure suggests a consistent gelation process and successful removal of volatile components, such as water and glycerol, during the heating process. The nanoparticle assembly forms a stable, interconnected network, which is likely to contribute to the material’s overall mechanical properties and its ability to resist external stresses. This well-defined pore structure is characteristic of silica-based gels, indicating the formation of a three-dimensional network after the removal of water and glycerol. The pores in the structure are interconnected, which can be attributed to the calcium ion-mediated crosslinking that occurred during the gel preparation. This crosslinking likely enhanced the structural integrity of the gel, allowing for a well-formed, robust nanoparticle network. The homogeneous pore size distribution further suggests that the gel structure is highly stable and capable of maintaining its integrity under varying conditions, which is crucial for applications in thermal insulation and other industrial uses requiring stable, porous materials.

## 3. Conclusions

This study proposes an innovative dual-modification strategy for fabricating high-performance silica xerogels through the synergistic combination of controlled calcium ion (Ca^2^⁺) concentration and glycerol incorporation in the sol-gel system. Physicochemical analyses reveal that controlled Ca^2+^ incorporation (optimal at 6 wt.%) accelerates gelation kinetics while establishing a hybrid network through ionic complexation and hydrogen bonding. The resulting xerogels achieve exceptional compressive strength (30.8 MPa) while maintaining ultralow thermal conductivity. When the Ca^2+^ content increased from 2% to 8%, the gel time decreased from 870 s to 535 s, which indicated that calcium ions could significantly promote the formation of the gel network. Unlike traditional ion crosslinking methods, the combined use of Ca^2+^ ions and glycerol can form a uniform three-dimensional crosslinking framework. The results of scanning electron microscopy indicated that the gel structure was intact and the element distribution was uniform, which provides a structural basis for the improvement of its mechanical properties. The decreasing BET surface area (177 → 130 m^2^/g) and stable pore volume (~75 cm^3^/g) suggest Ca^2^⁺-induced densification of micropores while preserving mesoporosity. TGA analysis confirmed minimal mass loss (<10 wt.%) below 400 °C, with 85–90 wt.% residue at 1000 °C, demonstrating exceptional thermal resistance. Ca^2^⁺ enhances the crosslinking density and structural strength by forming Si–O–Ca-O-Si bonds with Si–OH. Glycerol alleviates the drying shrinkage stress through hydrogen bonding, prevents cracking, and stabilizes the pore structure. The two work together to achieve the high strength and thermal stability of the dry gel. The synergistic effects of Ca^2^⁺ (network reinforcement) and glycerol (pore stabilization) enable a scalable, ambient-drying route to mechanically robust, thermally stable xerogels. This study provides a simple and effective method for synthesizing high-performance silica xerogels, highlighting their potential for a wide range of practical applications.

## 4. Materials and Methods

### 4.1. Materials

Silica sol (30 wt.%) was provided by Zhejiang Yuda Chemical (Shaoxing, China) Co., China. Calcium chloride (CaCl_2_, powder) was purchased from Sinopharm Chemical Reagent Co., Shanghai, China. Glycerol (C_3_H_8_O_3_, 99.0%) was purchased from Sinopharm Chemical Reagent Co., Shanghai, China. All chemicals were received and used without further purification. All experiments used deionized water.

### 4.2. Preparation of Silicon-Calcium Xerogel

**Figure 10:**
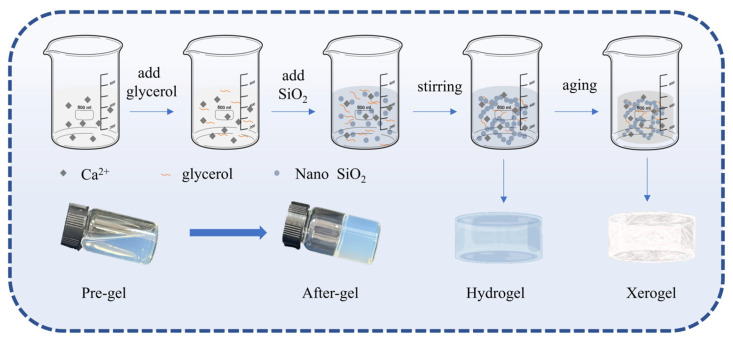
Preparation process of Silicon-Calcium xerogel.

Silica ceramic materials were prepared by the sol-gel method. Chemical compositions of silicon-calcium xerogel as shown in Table 2. With silica sol as the main material and calcium chloride as the curing agent, calcium chloride was dissolved in deionized water to form a calcium chloride solution with a 20 wt.% concentration. A certain amount of nano-silica sol was added with 5% glycerol. Aqueous solutions of 2%, 4%, 6%, and 8% calcium chloride and silica sol-glycerin solution were stirred in a magnetic mixer at room temperature with a speed of 500 rpm/min for 1 min. The bubbles were removed by ultrasound for 1 min. The mixed solution is placed in a circular plastic mold, aged at 45 °C for 3 days under atmospheric pressure. The preparation process as shown in Figure 10.

### 4.3. Characterization of Samples

The morphology of the silica matrix was observed by scanning electron microscopy (SEM, JSM7401, Shimadzu, Kyoto, Japan). The samples were coated with gold and observed with an accelerating voltage of 3.0 kV. The pore structure of the silica matrix was characterized by a nitrogen adsorption analyzer (Autosorb-1-C, Quantachrome, Boynton Beach, FL, USA) at liquid nitrogen temperature. The pore size distribution, the average pore diameter, and the pore volume were calculated by the Barrett-Joyner-Halenda (BJH) method based on the adsorption–desorption isotherms. The samples were characterized by XRD (XRD Ultima IV, Rigaku, Tokyo, Japan) for the crystalline phase identification. The diffraction intensity data were scanned with a sampling step of 0.02° in the 2θ range from 5° to 60°. Fourier transform infrared spectra (FTIR) were recorded at a resolution of 2 cm^−1^ and 32 scans using an FTIR spectrometer (VERTEX 80V, Bruker, Ettlingen, Germany) in the wavelength range 500–4000 cm^−1^. The dry samples were ground with KBr and then pressed into pellets. The thermal stability of the samples was analyzed using a TGA Q500 thermogravimetric analyzer (TA, New Castle, DE, USA). Thermogravimetric analysis was performed from 50 to 1000 °C at a heating rate of 10 °C/min under a nitrogen atmosphere. Compression tests were performed on a universal testing machine (Q500 from TA Company, New Castle, DE, USA) at room temperature, and then the stress–strain curves were recorded for each sample. In mechanics performance testing, three consecutive experiments were performed, and the data were expressed as the mean. Rheological properties of hydrogels were examined using Rotational rheometer (Rheowin MARS60, Rheowin MAS Company, Karlsruhe, Germany) at a temperature of 25 °C.

## Figures and Tables

**Table 1:** The specific surface area and pore volume of silicon-calcium xerogel.

Sample ID	Surface Area m^2^/g	Pore Volume cm^3^/g
SC-2	177.26	74.95
SC-4	162.22	74.66
SC-6	139.54	74.97
SC-8	130.80	75.15

**Table 2:** Chemical compositions of silicon-calcium xerogel.

Sample	Silicon Sol (g)	Glycerin (g)	Calcium Chloride Solution (g)	Calcium Ion Content (wt.%)
SC-2	20	0.3	0.6	2%
SC-4	20	0.3	1.2	4%
SC-6	20	0.3	1.8	6%
SC-8	20	0.3	2.4	8%

## Data Availability

Data are available from the authors. Samples of the compounds are available from the authors.
